# A Nutritional Profile of the Trap-Nesting Wasp *Trypoxylon lactitarse* (Hymenoptera: Crabronidae): Comparison of Sexes and Overwintering and Non-Overwintering Generations

**DOI:** 10.3390/insects8010003

**Published:** 2017-01-03

**Authors:** Timothy M. Judd, Matthew P. Fasnacht

**Affiliations:** 1 Department of Biology, Southeast Missouri State University, Cape Girardeau, MO 63701, USA; 2 Department of Chemistry, Southeast Missouri State University, Cape Girardeau, MO 63701, USA

**Keywords:** *Trypoxylon*, nutrition, wasp, micronutrient, macronutrient, bivoltine

## Abstract

The wasp *Trypoxylon lactitarse* Saussure is a bivoltine trap-nesting species that possesses a non-overwintering generation (G1) and a generation that overwinters as a prepupa (G2). Thus, the nutritional needs of the G1 individuals were predicted to be different than the G2 because the latter generation needs to store energy prior to diapause. Trap-nesting *Trypoxylon* are also of interest because, unlike most Hymenoptera, the males guard the nest while females forage. Thus, males may lose nutrients as they stay and guard the nest. In this study, a nutritional profile was created for *T. lactitarse* to compare the macronutrient (protein, carbohydrates, and lipids) and micronutrient (Ca, Cu, Fe, K, Mg, Mn, Na, and Zn) levels of the different life stages of the wasp and compare individuals of the G1 and G2 generations. There were distinct changes in the nutrient levels relative to the original food source as individuals metamorphosed into larvae, pupae, and adults. G1 larvae had higher levels of carbohydrates than G2 larvae. G2 larvae had higher levels of lipids and K than G1 larvae, indicating possible differences in energy storage. In adults, there was an increase in levels of carbohydrates and Mn. Parental males, which stay and guard the nest, were found to have higher levels of carbohydrates at the end of the nesting period than females and emerging adults. One possible implication is that females may feed males during the nesting period, as the females are the only individuals to forage.

## 1. Introduction

There has been a growing interest in understanding the biology of solitary wasps and bees from many fronts. Continued threats to honey bee health has created increased interest in understanding the biology of other native pollinators, including solitary bees and wasps [1]. Many solitary wasps are reared and used for biological pest management [2]. Newer hypotheses on the evolution of sociality, such as the diapause ground plan hypothesis [3,4] and the ovarian ground plan hypothesis [5], point to solitary ancestors that are either bivoltine or serial egg laying. Because nutrition plays a large role in caste determination in wasps and bees [6,7], a better understanding of the nutritional ecology of solitary species will provide better model systems to test the aforementioned models. Unfortunately, we know very little about the basic nutritional ecology of most solitary wasps and bees beyond the prey they collect. Understanding the nutritional ecology of solitary wasps and bees may help us understand the biology of natural pollinators and provide the necessary foundation to understand the role of nutrition in the evolution of social insects.

Wasps and bees that store food in cells provide excellent models for exploring nutritional ecology [8,9,10]. This is especially true for the trap-nest Hymenoptera. Trap-nesting wasps and bees build nests in preexisting cavities, where they produce a linear series of cells [11]. The name “trap-nest” refers to manmade cavities that wasps and bees readily use [11]. This system allows for easy collection and manipulation. Much of the nutritional ecology work on trap-nesting wasps and mud wasps examined the prey captured by the animals. A few studies looked at the food assimilation ability of the wasp [12] or the caloric value of the food [8,10,13], but there are fewer studies that examined the actual nutritional content of the food and individuals in these systems. The parent provides the offspring with its entire food stores for development and, thus, this results in a nutritionally closed system until the individual ecloses from pupation [9]. By measuring the nutritional content of food, what the parents provide is easily determined and, by measuring nutritional content in individuals, we can determine what nutrients are acquired and the changes in nutrient levels during development.

The trap-nesting wasp *Trypoxylon lactitarse* Saussure is found throughout North America and parts of South America [14]. *T. lactitarse* females provision their nests with spiders. Once a cell is full, the female lays an egg on one of the prey items and then seals the chamber with mud. Nest architecture and types of prey have been well documented throughout their range [11,15,16]. Unlike other trap-nest genera, *Trypoxylon* males guard the nest while the female forages [11,17,18,19,20,21,22,23,24,25]. The male’s guarding behavior was previously shown to reduce parasitism from other insects [13,19]. However, what actually encourages the male to stay and guard the nest is still not well understood.

In Missouri, *T. lactitarse* is bivoltine; one generation is produced during the summer (G1) and the second generation overwinters at the prepupa stage (G2). Adults emerge the following spring and continue the nesting cycle. Thus, in temperate climates, the nutritional needs of the G1 generation may differ from the G2 generation. We predicted that there would be differences in the nutritional content between the immatures (larvae and pupae) of G1 and G2. Specifically, we expected higher levels of nutrients associated with storage, such as lipids, in the G2 generation. In addition to generational comparisons, changes in nutritional levels of adults at eclosion to those completing a nest were also examined. Males rarely leave the nest, thus it would be of interest to examine what happens to their nutritional levels from eclosion to the end of a nest. Females are able to forage, and we would predict that their nutrient levels should be more constant than the nutrient levels of males. However, a drop in nutritional levels in females would indicate that they allocate more time and energy into foraging for prey than for their own nourishment.

In this study, we created a nutritional profile for *Trypoxylon lactitarse*. Individual eggs, larvae pupae, emerging adults, and nesting adults were analyzed for their macro- and micronutrient content. This profile allowed us to determine if there are nutritional differences between individuals from the G1 and G2 generations, specifically the larvae. We also compared changes between stages of development and during adulthood. Finally, we were able to determine if there were any differences between males and females.

## 2. Materials and Methods

### 2.1. Trap-Nests

The trap-nests were modified from the original design created by Krombein [11]. Each trap-nest had two 1.05 or 1.35 cm sized holes that allowed us to slide a paper tube into the trap (Supplementary Materials, Figure S1). To access the trap contents, only the paper tube needed to be removed. The complete design of the trap-nests is described in detail in the Supplementary Materials, Methods S1.

### 2.2. Study Sites

A total of 115 trap-nests were placed in four sites in Missouri in order to insure a large enough sample size without eliminating the populations [11]: Saint Francois State Park (Saint Francois, MO, USA), Juden Creek Nature Preserve (Cape Girardeau, MO, USA), Robertsville State Park (Robertsville, MO, USA), and Voelkerding Slough (Dutzow, MO, USA). The nests were placed on dead or living tree limbs along the edge of an open field. Each site had 15 trap-nests of each size except Juden Creek, which had 25 of the 1.35 cm trap-nests, and Robertsville, which did not have 1.05 cm trap-nests.

### 2.3. Monitoring and Field Collections

The trap-nests were checked two to three times a week for occupation. *Trypoxylon* is the only genus of trap-nest Hymenoptera in Missouri in which the male guards the nest. Thus, it was easy to distinguish if the nest was occupied by *Trypoxylon. Trypoxylon lactitarse* is the only species of *Trypoxylon* in Southeast Missouri that nests in cavity sizes used in this study. Other species of *Trypoxylon* use smaller cavities [11]. When both paper tubes of the trap-nest unit were filled with nests, one was removed for investigation and the other one was left to prevent a population damage. [11]. A fresh paper tube was placed into the empty hole where a paper tube was removed.

If a nest was encountered that was almost filled (one tube completed and the other almost completed), the male and female were captured by either plugging the tube when both adults were inside or placing a 5 mL centrifuge tube over the hole and waiting until the female backed into the tube, then capping the paper tube to trap the male. The adults were placed in ice in a cooler in the field and then transferred to a −80 °C freezer in the laboratory.

### 2.4. Opening Tubes and Rearing

The tubes were opened slowly to reveal individual cells. If eggs were collected, they were removed from the brood cell containing spiders and then stored in a microcentrifuge tube separate from the food at −80 °C. Only the final larval stage was used in this study. Larvae that were not fully grown when collected from the field were placed in 12-well flat-bottom cell culture plates (well volume of 6 mL) with the remaining food from their cell and reared until all of the food was eaten. Fully developed larvae were frozen in individually labeled tubes at −80 °C. Experience showed us that the larvae were unable to build a cocoon in the plates. In order to allow larvae to pupate, they were left in the paper tube. Once the cocoon was formed, the cocoon was removed from the tube and placed in a culture plate. Unlike most trap-nesting wasps and bees, *Trypoxylon* does not position the females in brood cells located in the back and males in brood cells in the front part of the nest [11], and no distinguishing features were found to determine the sex of larvae. Pupae were collected by monitoring certain individuals that had a portion of the casing removed. Once they metamorphosed, other individuals collected at a similar time were checked. Pupae were sexed by counting the number of segments on the abdomen and counting segments on the antennae. Some pupal cells were reared to adults; once an adult emerged, the wasp was frozen at −80 °C in an individually labeled tube. In all cases, trays were checked daily.

### 2.5. Measurements

In total, half the individuals were designated for the macronutrient analysis and the other half for the micronutrient analysis. All adults had their head width (HW), pronotum width (PW), and wing length (WL) measured as a correlation to size, and had their wet mass (WM) determined. HW was measured for larvae and the HW and PW were determined for pupae. In all individuals, the HW was measured just posterior to the eye. Individuals designated for the micronutrient analysis were dried to constant mass at 40 °C and then measured for dry mass (DM).

### 2.6. Micronutrient Analysis

Individuals were measured for levels of calcium (Ca), copper (Cu), Iron (Fe), potassium (K), magnesium (MG), manganese (Mn), sodium (Na), and zinc (Zn) using inductively coupled plasma optical emission spectrometry (ICP-OES, Perkin Elmer Optima 3000 DV, PerkinElmer, Waltham, MA, USA). The samples were dissolved in trace metal-grade nitric acid and measured in the same manner outlined in Judd et al. [26], including the use of scandium as an internal standard.

### 2.7. Macronutrient Analyses

Levels of proteins, carbohydrates, and lipids were determined using the Bradford assay, anthrone assay, and phosphovanillan assay, respectively. Samples were prepared and analyzed in the same manner as Judd et al. [26], such that all three nutrients were measured from each individual. The samples were analyzed using a spectrophotometer (Beckmann DU^®^ 730, Beckman Coulter Inc., Brea, CA, USA). Samples that approached the peak absorbance were diluted and reanalyzed.

### 2.8. Data Analysis

The levels of macronutrients or micronutrients for individuals were divided by the WM or DM, respectively, to allow for cross-life-stage comparisons. Hereafter, “level” of a nutrient refers to level per unit mass.

#### 2.8.1. Life Stage Comparisons

In all analyses comparing life stages, macronutrients were analyzed separately from micronutrients. Separate analyses were performed for food, eggs, immatures (larvae and pupae), and adults. For the egg and food analyses, the generations (G1 and G2) were compared to the levels of nutrients using a Mann-Whitney U test followed by a Bonferroni table-wide correction [27]. In the immature and adult analyses, the data were log transformed. A new categorical variable was created that combined the generation, life stage, and sex of the individuals. This variable was then compared to the nutrient levels using a MANOVA followed by a Tukey honest significant difference test (TSHD) (SAS/STAT Software, SAS Institute Inc., Cary, NC, USA). A similar procedure was done by Ashcraft and Judd [28]. This new variable allowed us to compare larvae (where the sex of the individuals was not known) with pupae (in which the sex was determined). In the adult analysis, the individuals collected at the end of nesting (hereafter referred to as old individuals) were combined due to small sample sizes. The new variable allowed the comparison of the combined old individuals with the emerged adults from the G1 and G2 generations.

#### 2.8.2. Changes during Development

There is no additional food intake during the development period, other than what is available in the cell. Hence, we also compared the changes in levels of nutrients relative to the food plus egg nutrients during development. The mean level of each nutrient per unit mass of the food plus the egg was determined for the G1 and G2 generations, hereafter called the “average food value” (AFV). The percent change of each nutrient (percent change in nutrient: PCN) relative to the food was determined using the AFV for G1 and G2 for larvae, male and female pupae, and emerging male and female adults. The results were analyzed in two ways. Individual PCN values were compared with a principle components analysis (PCA) to examine how nutrients change in the different castes. In order to examine all the nutrients in a single analysis, the average PCN value was determined for each nutrient for each life stage. Because the PCN values are negative or positive, relative to the food, the data set is similar to that of a microarray. A hierarchical cluster analysis (HCA) using GENESIS [29] was performed to compare changes in all the nutrients.

#### 2.8.3. Morphology and Nutrient Comparisons

Generalized linear models were used to determine if larval head width correlated with nutrient levels per unit mass. Separate analyses for macronutrients and micronutrients were performed (SAS/STAT Software, SAS Institute Inc., Cary, NC, USA). Pupae and adults had multiple morphological features measured; thus, for each analysis, two PCAs were run—one for morphological features (HW and PW for pupae and HW, PW, and WL for adults) and another for nutrient levels per unit mass. PCAs were followed by a canonical correlation analysis (CCA) to determine any correlation between size and nutrient levels (SAS/Stat Software). Four analyses were performed. Males and females were each run separately, and macro- and micronutrients were run separately.

## 3. Results

### 3.1. Eggs and Food

The results for the analyses for eggs and food are detailed in the Supplementary Materials, Results S1 (Figures S1 and S2, Table S1). In summary, no significant differences were found between any macronutrient, caloric values, or detectable micronutrient in the eggs (Figure S2). The only detectable difference between the food collected during G1 and food collected from G2 were that protein levels in the food from the G2 nests were significantly higher than levels in food from G1 nests (T = 4, *p* < 0.01, Figure S3a).

### 3.2. Immatures

#### 3.2.1. Macronutrients

**Figure 1:**
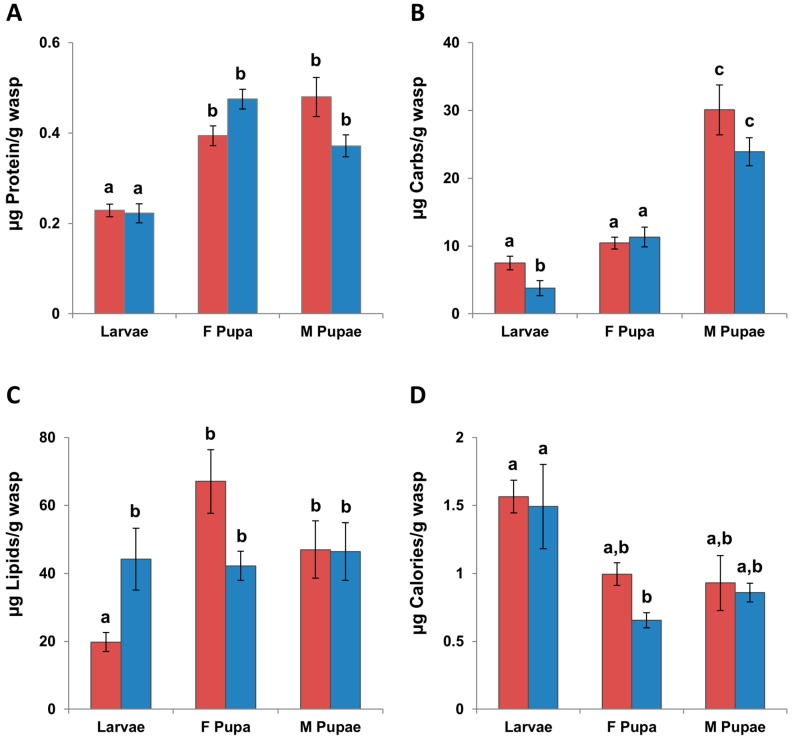
The mean levels of (**A**) protein; (**B**) carbohydrates; (**C**) lipids; and (**D**) calories per unit wet mass in fifth instar larvae (N_G1_ = 8, N_G2_ = 7), and male (N_G1_ = 7, N_G2_ = 4) and female (N_G1_ = 8, N_G2_ = 6) pupae of *Trypoxylon lactitarse* from the G1 (red) and G2 (blue) generations. Error bars indicate standard error. For each graph, different letters (a–c) indicate significant differences between groups (MANOVA followed by Tukey’s honest significant difference test (THSD)).

The overall MANOVA for the macronutrients in immatures was significant (*F* = 21.73, df = 3, 11, *p* < 0.0001) and significant for each nutrient (Table 1). There was no significant difference between the levels of protein in larvae from G1 and G2 or between the G1 and G2 male and female pupae. All of the pupae stages had higher levels of protein than the G1 and G2 larvae (*p* < 0.009, THSD, Figure 1A).

G1 larvae had significantly higher levels of carbohydrates (*p* = 0.01, THSD, Figure 1B) and significantly lower levels of lipids (*p* = 0.0022, THSD, Figure 1C) than G2 larvae. Male pupae had higher levels of carbohydrates than female pupae and larvae (*p* < 0.018, THSD). Female G1 and G2 pupae had higher levels of carbohydrates than G2 larvae (*p* < 0.0001, THSD), but not higher than G1 larvae (Figure 1B). There were no significant differences between levels of lipids in pupae and the G2 larvae, but all pupae and G2 larvae had higher levels of lipids than G1 larvae (*p* < 0.015, THSD, Figure 1C).

#### 3.2.2. Calories

The overall ANOVA for the calories in immatures was significant (*F* = 4.30, df = 5, 34, THSD, *p* = 0.0039). The G2 female pupae had lower caloric levels than both G1 and G2 larvae (Figure 1D). There were no other significant differences between the caloric levels of the immature stages.

#### 3.2.3. Micronutrients

**Figure 2:**
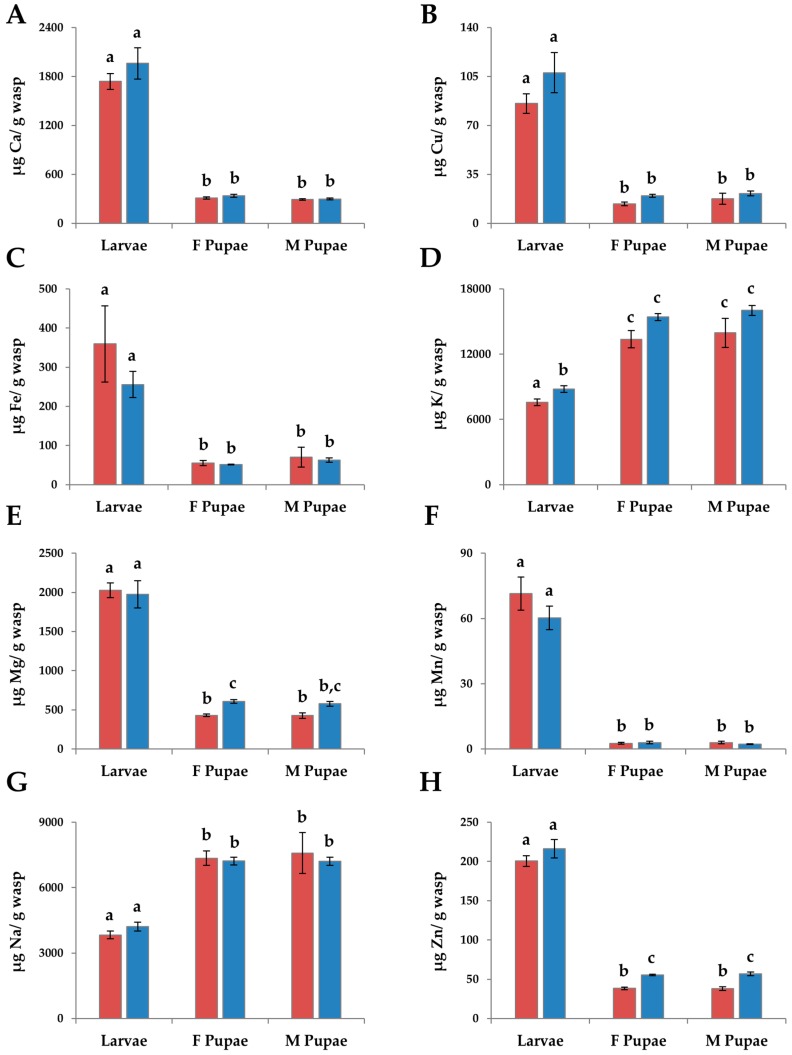
The mean levels of (**A**) Ca; (**B**) Cu; (**C**) Fe; (**D**) K; (**E**) Mg; (**F**) Mn; (**G**) Na; and (**H**) Zn per unit dry mass in fifth instar larvae (N_G1_ = 10, N_G2_ = 10), and male (N_G1_ = 4, N_G2_ = 6) and female (N_G1_ = 5, N_G2_ = 10) pupae of *Trypoxylon lactitarse* from the G1 and G2 generations. Error bars indicate standard error. For each graph, different letters (a–c) indicate significant differences between groups (MANOVA followed by THSD).

The overall MANOVA for the micronutrients in immatures was significant (*F* = 14.24, df = 40, 142.28, *p* < 0.0001) and significant for each individual nutrient (Table 1). In all elements, except K and Na, larvae had higher levels of the elements than pupae (*p* < 0.0001, THSD, Figure 2A–C,E,F,H). Levels of K and Na were significantly higher in pupae than in larvae (*p* < 0.0001, THSD, Figure 2D,G). There were no significant differences between G1 larvae and G2 larvae in all elements except K. G2 larvae had significantly higher levels of K than G1 larvae (*p* < 0.05, THSD, Figure 2D). There was no significant difference among the pupae for all elements except Mg and Zn. G2 female pupae had significantly higher levels of Mg than G1 male pupae (*p* = 0.02, THSD) and G1 female pupae (*p* = 0.016, THSD, Figure 2E). G2 pupae had significantly higher levels of Zn than G1 pupae (*p* < 0.0001, THSD, Figure 2H).

### 3.3. Changes in Individuals from Initial Nutrients

**Figure 3:**
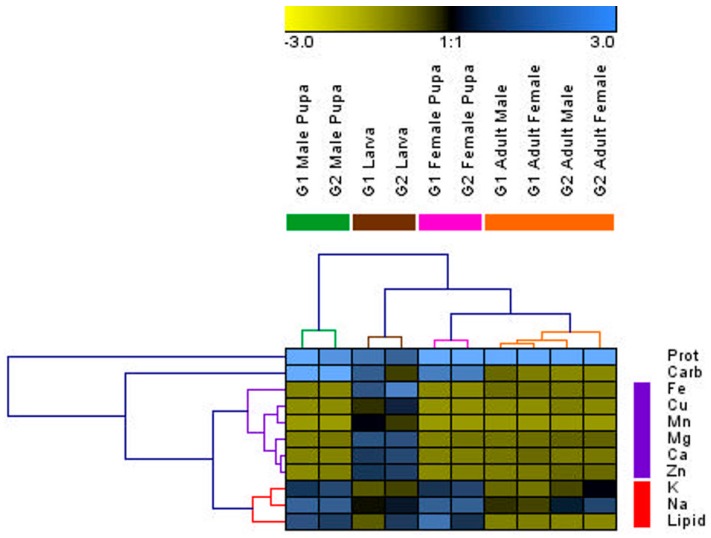
Mean percent change of nutrients relative to initial nutrients (PCN). Increases in PCN are indicated by levels of blue and decreases in PCN are indicated by yellow. Results for the hierarchal analysis are shown for the nutrients and the life stages.

The HCA produced two distinct clusters of nutrients (Figure 3): one containing Na, K, and lipids and another containing the rest of the micronutrients. Carbohydrates and proteins fell outside these clusters. Na, K, and lipids had positive PCN scores during pupation and negative scores after emergence. The other micronutrients showed positive PCN scores during the larval period and then negative scores in pupation through emergence. Carbohydrates remained positive throughout development but became negative during emergence, whereas the PCN score for protein was high throughout (Figure 3). The PCA analyses showed very similar results to the HCA analysis; the plots and eigenvectors (Tables S2–S5) can be found in the Supplementary Materials, Results S2. The three macronutrients showed different trajectories in the macronutrient PCA analysis. Male pupae separated from the rest of the life stages based on PCN scores for carbohydrates and pupae separated from the other life stages due to lower PCN scores for protein (Figure S4). Thus, the separation of male pupae in the HCA (Figure 3) was probably driven by the levels of carbohydrates. Indeed, if carbohydrates are removed from the analysis, the male and female pupae cluster together (Figure S5). In the micronutrient PCA, the larvae separated from the other life stages because they had very different PCN scores (Figure 3 and Figure S6). The primary division between pupae and adults was due to the differences in levels of K and Na (Figure S6). G2 adult females had more similar PCN scores to pupae, explaining why this life stage was basal in the adult cluster (Figure 3).

### 3.4. Adults

The overall MANOVA’s for the macronutrients (*F* = 6.79, df = 15, 86, *p* < 0.0001) and micronutrients (*F* = 14.35, df = 40, 203.3, *p* < 0.0001) were significant. Of all the nutrients, only Fe did not show any significant differences among the adult life stages (Table 2). The overall ANOVA for the calories in adults was also significant (*F* = 8.70, df = 5, 33, *p* < 0.0001).

#### 3.4.1. Changes in Age

**Figure 4:**
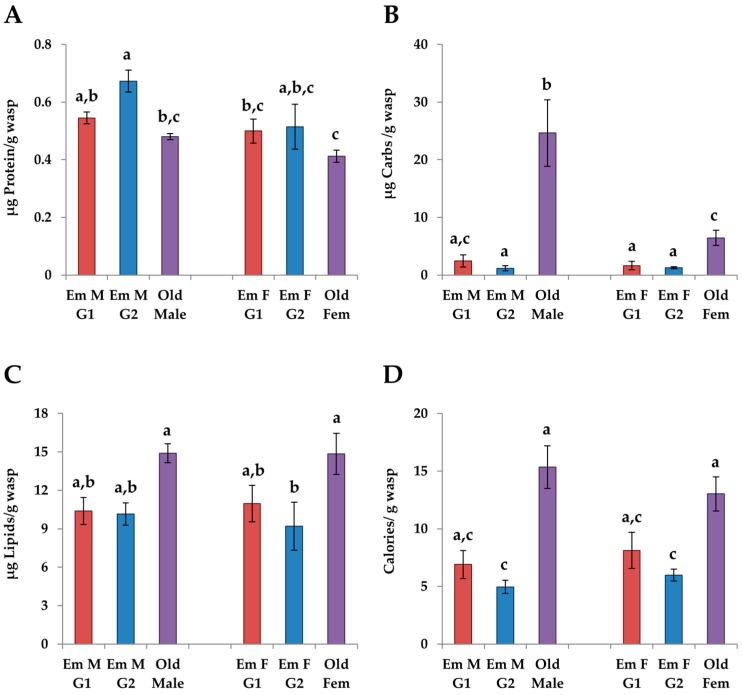
The mean levels of macronutrients: (**A**) protein; (**B**) carbohydrates; (**C**) lipids; and (**D**) calories per unit wet mass in eclosing males (N_G1_ = 6, N_G2_ = 4) and females (N_G1_ = 6, N_G2_ = 5) from the G1 and G2 generations and old adults (both generations combined, N_Males_ = 8, N_Females_ = 10) of *Trypoxylon lactitarse*. Error bars indicate standard error. For each graph, different letters (a–c) indicate significant differences between groups (MANOVA followed by THSD).

**Figure 5:**
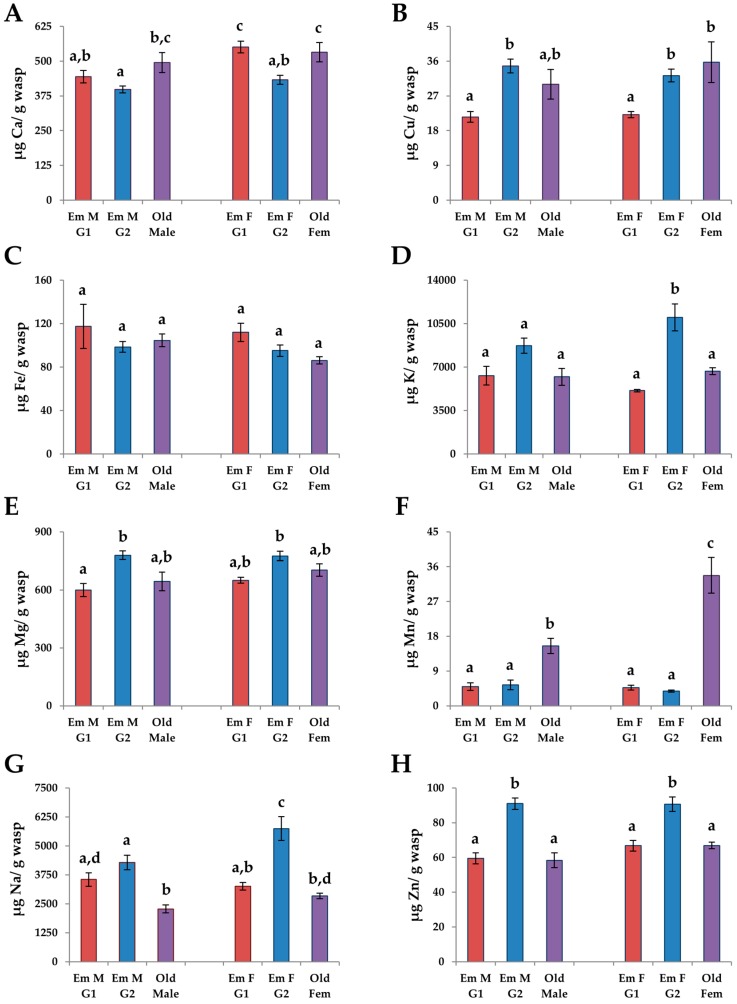
The mean levels of micronutrients: (**A**) Ca; (**B**) Cu; (**C**) Fe; (**D**) K; (**E**) Mg; (**F**) Mn; (**G**) Na; and (**H**) Zn per unit dry mass in eclosing males (N_G1_ = 9, N_G2_ = 17) and females (N_G1_ = 6, N_G2_ = 16) from the G1 and G2 generations and old adults (both generations combined, N_Males_ = 5, N_Females_ = 6) of *Trypoxylon lactitarse*. Error bars indicate standard error. For each graph, different letters (a–c) indicate significant differences between groups (MANOVA followed by THSD).

The levels of carbohydrates were significantly lower in emerging individuals than old individuals in both sexes (females: *p*_G1_ = 0.049, *p*_G2_ = 0.054; males: *p* < 0.0001 for both, THSD, Figure 4B). Old females had significantly higher levels of lipids than G2 emerging females (Figure 4C), while protein levels in males were significantly higher in G2 emerging males than old males (*p* = 0.0095, THSD, Figure 5A). The caloric value was higher in old individuals than G2 emerging individuals in both sexes (females: *p* = 0.001; males: *p* = 0.0054, THSD, Figure 4D) but the caloric values were only higher than G1 emerging individuals in females (*p* = 0.04).

Levels of Mn were higher in old individuals than in either G1 or G2 emerging individuals in both sexes (*p* < 0.0001 for all, THSD, Figure 5F). Levels of Ca were higher in old individuals than G2 emerging individuals (females: *p* = 0.048; males: *p* = 0.053, THSD, Figure 5A) while levels of Na were significantly higher in G1 and G2 emerging individuals (females: *p*_G1_ = 0.039, *p*_G2_ < 0.0001; males: *p*_G1_ = 0.039, *p*_G2_ =0.025, THSD, Figure 5G). Levels of Zn were significantly higher in G2 emerging individuals than old individuals (females: *p* = 0.048; males: *p* < 0.0001, THSD, Figure 5H). Unlike males, levels of Cu were higher in old females than G1 emerging females (*p* = 0.018; THSD, Figure 5B) and levels of K were higher in G2 emerging females than old females (*p* = 0.0081, THSD, Figure 5D).

#### 3.4.2. G1 vs. G2 Emerging Adults

In both males and females, G1 emerging adults had lower levels of Cu than G2 emerging males and females, respectively (*p*_m_ < 0.0001, *p*_f_ = 0.016, THSD, Figure 5B). G2 emerging males and females had higher levels of K (*p*_m_ = 0.053, *p*_f_ < 0.0001, THSD) and Zn (*p*_m_ < 0.0001, *p*_f_ = 0.0059, THSD) per unit mass than G1 emerging males and females, respectively (Figure 5D,H). There were only a few differences between the sexes when comparing G1 and G2 emerging adults. G1 emerging females had higher levels of Ca than G2 emerging females (*p* = 0.0092, THSD, Figure 5A), while levels of Na were higher in G2 emerging females (*p* = 0.001, THSD, Figure 5G). G2 emerging males had higher levels of Mg than G1 emerging males (*p* = 0.012, THSD, Figure 5E).

#### 3.4.3. Males vs. Females

Old males had significantly higher levels of carbohydrates than old females (*p* = 0.0017, THSD, Figure 4B), while old females had significantly higher levels of Mn (*p* = 0.012, THSD, Figure 5F). There were very few differences between emerging males and females. G1 females had higher levels of Ca than G1 emerging males (*p* = 0.053, THSD, Figure 5A) and G2 females had significantly higher levels of Na than G2 emerging males (*p* = 0.039, THSD, Figure 5G).

### 3.5. Size and Nutrient Relationships

#### 3.5.1. Larvae

There was no significant correlation between larval head width and levels of macronutrients (*F* = 1.62, df = 15, 14.2, *p* = 0.18) or micronutrients (*F* = 0.22, df = 16, 20, *p* = 0.23).

#### 3.5.2. Pupae

Females showed a positive relationship between head width and lipid levels (*F* = 27.39, df = 9, 36.7, CCA, *p* < 0.0001). In the male pupal analysis, both head width and pronotum width were correlated (*F* = 11.81, df = 9, 29.36, CCA, *p* < 0.0001). As male head width increased, levels of carbohydrates increased and levels of protein decreased. In addition, as male pronotum width increased, lipid levels increased and protein levels decreased. Tables showing the results of the CCA analysis can be found in Supplementary Materials Results S3 (Tables S6–S12).

There was no significant correlation between micronutrient levels and size in females (*F* = 0.49, df_Num_ = 24, df_Den_ =6.4, CCA, *p* = 0.9) or males (*F* = 2.5, df = 24, 3.5, CCA, *p* = 0.21).

#### 3.5.3. Adults

The CCA was significant for size and macronutrient comparisons for females (*F* = 27.39, df = 9, 36.7, CCA, *p* < 0.0001) and males (*F* = 11.81, df = 9, 29.36, CCA, *p* < 0.0001). Tables showing the results of the CCA analysis can be found in Supplementary Materials Results S3 (Tables S13–S19). Both sexes showed a strong positive relationship between head width and levels of carbohydrates. Females showed a negative relationship between wing length and levels of lipids and proteins. Therefore, larger females had higher levels of carbohydrates and lower levels of protein and lipids. Males showed a negative relationship between both pronotum width and wing length with levels of protein. Larger males had higher levels of carbohydrates and lower levels of protein.

As with the immatures, there was no significant correlation between micronutrient levels and size in females (*F* = 0.49, df = 24, 6.4, CCA, *p* = 0.9) or males (*F* = 2.5, df = 24, 3.5, CCA, *p* = 0.21).

## 4. Discussion

### 4.1. Changes During Development

The meta-analyses (HCA and PCA) revealed several major changes in the nutritional profile of individuals during development. First, during pupation, there was a loss of most micronutrients, except for K and Na, relative to the amount that existed in the food. These nutrients may have been lost during the molting process, as the individuals retain their meconium until eclosion. Arthropods have been shown to use the molting process to remove certain metals [30,31]. The second emerging pattern was levels of K and Na seeming to correlate with the levels of lipids, which matches what was observed in the individual analyses. It is during the transition from pupa to adult that there was a reduction of carbohydrates and lipids. As both of these nutrients are involved in energy storage, the reduction is probably due to the metabolic needs of this metamorphosis.

*Trypoxylon lactitarse* overwinters at the prepupal stage; therefore, the differences between G1 and G2 larvae gives us insight into the differences between individuals that will overwinter versus those that will continue to develop within a season. G1 larvae had higher levels of carbohydrates than G2 larvae but had lower lipid levels. This difference makes biological sense because G1 larvae proceed directly into pupation; thus, there is no need for large amounts of stored energy. G2 larvae do not pupate until the spring; therefore, lipid stores would be necessary to overwinter. G2 immatures had higher levels of K than G1 immatures. A similar pattern was observed in *Polistes metricus* Say [26]. Reproductive-destined immatures had higher levels of lipids and K than worker-destined immatures. The high K levels were retained through eclosion in adult *P. metricus* and subsequently lost as adults matured. A similar pattern was observed in *T. lactitarse* in this study. Jungreis and Tojo [32] found that nitrogenous waste from metamorphosis is accumulated in the fat body in the form of potassium urate in the silk worm, *Hyalophora cecropia* L., and the function of the Malpighian tubules is arrested during metamorphosis. Overwintering individuals may have higher levels of potassium to allow for more nitrogenous waste storage. If this is true, then there should be higher levels of uric acid found in G2 larvae than G1 larvae. The levels of potassium decreased during the lifetime of adults.

Based on the meta-analyses, levels of Na track the levels of lipids and K. Although the individual analysis did not detect significant differences between G1 and G2 larvae, the results of the meta-analyses suggests that Na was higher in G2. Na is a common ice-nucleating agent in overwintering insects [33,34,35]. Thus, a small rise in Na levels in G2 larvae would be consistent with the fact that they overwinter.

### 4.2. Changes in Adults

Levels of Mn were low in eclosing adults and levels increased markedly in females versus those in males. Increases in Mn levels were observed in adult males and females of *Polistes metricus* [26] and in workers of *Temnothorax curvispinosus* Mayr during periods of foraging [36]. There is some indication that increased levels of Mn are associated with an increase in taste perception [37] and foraging behavior [38]. The levels of Mn are related to the activation of *malvolio*, a Mn transporter that is higher in foraging honeybees [38]. In *P. metricus* and honeybees, foraging individuals have higher levels of Mn than those that do not [26,38], and foraging individuals have equal levels of *malvolio* expression [38,39]. In contrast to females, male *T. lactitarse* do not forage. The levels of Mn are consistent with this behavior and would predict higher *malvolio* expression in females than in males in *T. lactitarse*. If this connection is supported, then this phenomenon may be consistent throughout Hymenoptera.

One striking result when comparing adults were the high levels of carbohydrates in males. Males start their adult life searching for and competing for nest sites. This could be energetically costly. Once a nest is obtained, males stay in the nest and have not been known to forage [17,18,19,22,40,41,42]. Females, which have access to flowers, have been observed foraging for nectar in addition to prey items [43]. The result suggests that males are potentially obtaining carbohydrates from females. Paetzel [19] observed females and males of *Trypoxylon rubrocinctum* Packard, which have similar male guarding-behavior as *T. lactitarse* “…*antennal waving and mouth clashing*…”, which could be trophallaxis. Rau [20] observed trophallaxis between the female and male of *T. clavatum* Say. Hook and Matthews [13] suggested that males could possibly be induced to guard the nest, but the mechanism was elusive. This study provides additional evidence for the possible mechanism; females feed males, and this behavior keeps males at the nest. If this is true, the females would benefit because the presence of males increases the chances that nests will not be parasitized. The males would benefit because the females replenish their nutritional stores, allowing them to renest quickly. The possibility that the males are being fed by the females in order to retain them at the nest is an intriguing mechanism that would promote the evolution of male nest-guarding in the *Trypoxylon* genus.

### 4.3. Size and Nutrient Levels

There was a definite relationship between size and nutrient levels in *T. lactitarse.* Larger pupae tended to have relatively larger stores of lipids during pupation. This relationship was lost after emergence; however, larger emerging adults had larger stores of carbohydrates. Larger male pupae had higher levels of carbohydrates per unit mass, but this relationship was not seen in female pupae. Together, these results suggest that the pupae are preferentially using the lipid stores during ecdysis to an adult. Smaller females may be using a higher percentage of their carbohydrate stores than larger females to fuel this stage of development. The wasps may also be converting some of the protein to carbohydrates because the level of lipids was lower in larger individuals. Gluconeogenesis from amino acids has been observed in insects when lipid levels are low [44,45,46].

### 4.4. G1 and G2 Generations and Social Insect Castes

There were distinct differences in the development of G1 and G2 individuals that reflect the nutritional needs for non-diapause and diapause strategies. This suggests that the cue to undergo diapause must occur early in the development of the wasp. Hunt [4] predicted that workers and gynes were derived from G1 and G2 generations of a solitary wasp, respectively. Although *Polistes* and *Trypoxylon* are not extremely phylogenetically close within Aculeata [47], the similarities between the worker- and gyne-destined larvae of *P. metricus* and the G1 and G2 larvae of *T. lactitarse*, respectively, are striking. Several studies have pointed to the fact that cues during larval development can influence caste development in social wasps such as *Polistes* [6,48,49,50,51]. Here, we see some nutritional differences that *Polistes* castes and G1 and G2 generations of *Trypoxylon* share. It would be of interest to determine if the cues that cause these differences are similar in both species and if they are shared among other species of Aculeata.

## Figures and Tables

**Table 1:** Results for each nutrient from the MANOVAs for macro- and micronutrients for larvae and pupae of *Trypoxylon lactitarse.*

Nutrient	df	*F*	*p*
Macronutrient Analysis			
Protein	5	21.76	*p* < 0.0001
Carbohydrates	5	31.29	*p* < 0.0001
Lipid	5	7.92	*p* < 0.0001
Micronutrient Analysis			
Ca	5	183.43	*p* < 0.0001
Cu	5	80.91	*p* < 0.0001
Fe	5	32.79	*p* < 0.0001
K	5	68.79	*p* < 0.0001
Mg	5	120.71	*p* < 0.0001
Mn	5	232.26	*p* < 0.0001
Na	5	45.60	*p* < 0.0001
Zn	5	344.00	*p* < 0.0001

**Table 2:** Results for each nutrient from the MANOVAs for macro- and micronutrients for adult *Trypoxylon lactitarse.*

Nutrient	df	*F*	*p*
Macronutrient Analysis			
Protein	5	6.27	*p* = 0.0003
Carbohydrates	5	15.30	*p* < 0.0001
Lipid	5	4.13	*p* = 0.005
Micronutrient Analysis			
Ca	5	7.16	*p* < 0.0001
Cu	5	8.22	*p* < 0.0001
Fe	5	0.93	*p* = 0.47
K	5	8.57	*p* < 0.0001
Mg	5	7.23	*p* < 0.0001
Mn	5	36.44	*p* < 0.0001

## Supplementary Materials

The following are available online at http://www.mdpi.com/2075-4450/8/1/3/s1, Methods S1: Trap-nests, Results S1: Eggs and Food Data, Results S2: PCA Results; Results S3: CCA Results.
