# Supplementary material for: A Nutritional Profile of the Trap-Nesting Wasp Trypoxylon lactitarse (Hymenoptera: Crabronidae): Comparison of Sexes and Overwintering and Non-Overwintering Generations
Source: Insects. 2017 Jan 3;8(1):3. doi: 10.3390/insects8010003 (PMC5371931; doi:10.3390/insects8010003)
Supplement: Supplementary file 1 [file insects-08-00003-s001.pdf]

Supplementary Materials: A Nutritional Profile of the Trap-Nesting Wasp *Trypoxylon lactitarse* (Hymenoptera: Crabronidae): Comparison of Sexes and Overwintering and Non-Overwintering Generations

Timothy M. Judd and Matthew P. Fasnacht

Methods S1: Trap-Nests

Two 16.5 × 2.5 × 2.5 cm trap-nests were created from wood with either a 1.05 or 1.35 cm sized hole drilled 14 cm into the trap-nest (Figure S1A). A scroll saw was used to create an arced cut across the trap-nest so that the first fifth of the nest could be removed. A paper tube (1.0 or 1.3 cm inner diameter) that fit into the hole was pushed in and the extra portion was cut so that the tube was flush with the edge of the trap (Figure S1A). The back end of the tube was capped with a small 0.5 cm thick piece of wooden dowel to prevent contents of a nest spilling into the back of the nest when the paper tube was removed. The front portion of the trap-nest was held in place with industrial strength Velcro® straps. Two nests were sandwiched between two pieces of 16.5 × 2.5 × 0.6 cm plywood and the whole unit was strapped together with two zip strips (Figure S1B).

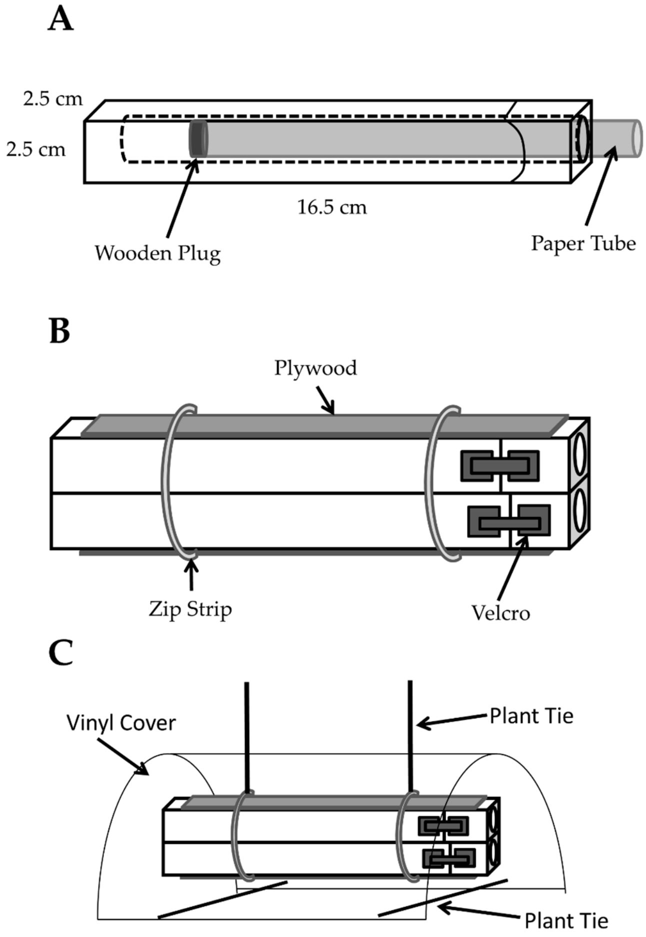

Figure S1. The modified trap-nests. (**A**) An individual block showing the scroll cut, paper tube, and wooden plug. The tube would be pushed into the hole so that it was flush with the outer surface; (**B**) two blocks strapped together between plywood strips; (**C**) the assembly hung inside a vinyl cover. Plant ties were used to hang the trap-nest and hold the bottom of the "tent" together.

The trap-nest unit was covered by a curved tent consisting of a 17.8 × 20.3 cm piece of vinyl flashing (beige or white) and held in pace by plant ties. Two ties extended from the top of the trap-nest unit through the flashing and were used to attach the trap to a branch. The other two were tied at the bottom to create the tent-like shape (Figure S1C).

Results S1: Eggs and Food

RS1.1 Eggs

No significant differences were found between the macronutrient and caloric levels of G1 and G2 eggs (Figure S2A–D). Of the micronutrients, only Ca, Fe, K, and Na had levels above the detection limit of the ICP-OES instrument. After analyzing the levels of these elements and the levels of macronutrients, we found no significant differences between the nutrient levels of G1 and G2 eggs (Figure S2E–H).

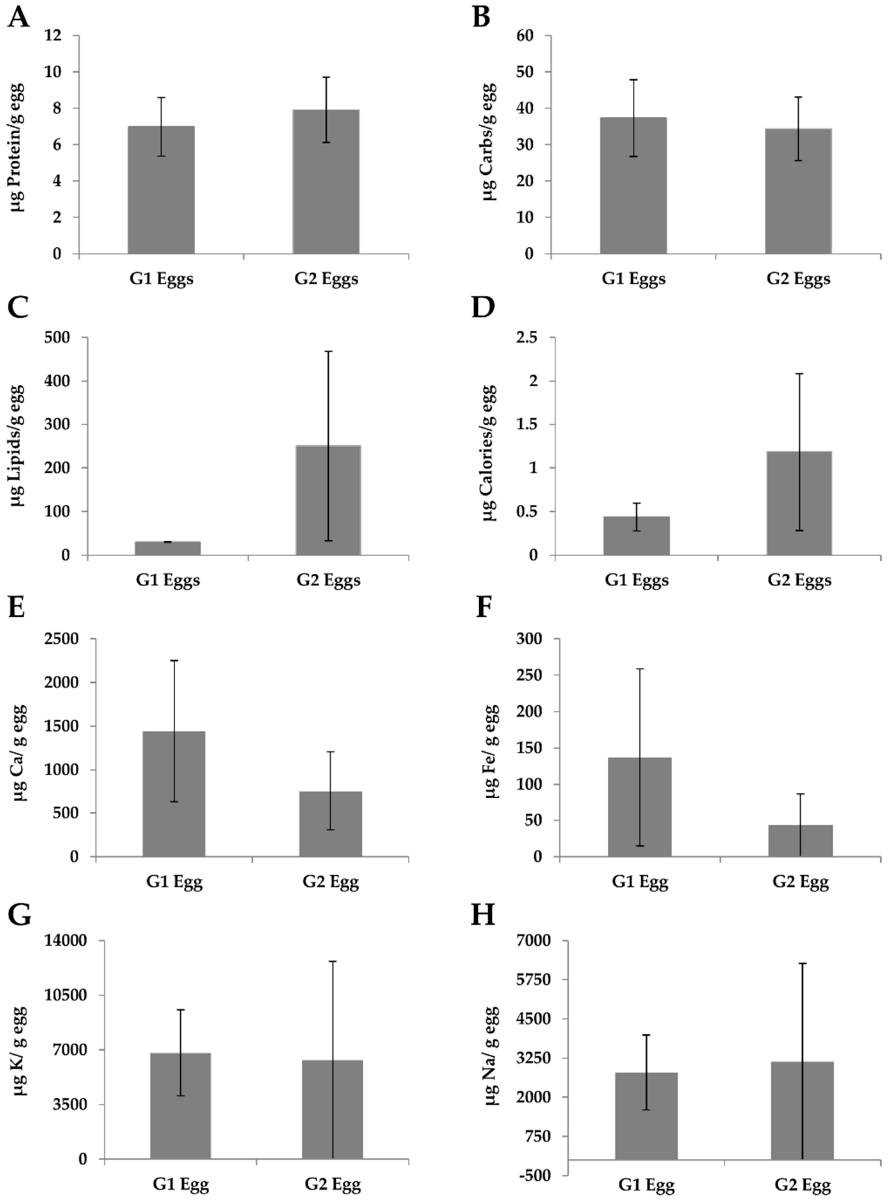

Figure S2. The mean levels of nutrients found in *Trypoxylon lactitarse* eggs from the G1 and G2 generations. Levels of (**A**) protein; (**B**) carbohydrates; (**C**) lipids; and (**D**) calories are reported per unit wet mass ($N_{G1} = 3$, $N_{G2} = 4$); and levels of (**E**) Ca; (**F**) Fe; (**G**) K; and (**H**) Na are reported per dry mass ($N_{G1} = 4$, $N_{G2} = 2$). Error bars indicate standard error. There were no significant differences between G1 and G2 eggs for any nutrient.

RS1.2 Food

There was a significantly higher level of protein in the food from the G2 nests than the G1 nests ($T = 4$, $p < 0.01$, Figure S3A). There were no significant differences between the levels of carbohydrates, lipids, or calories in the food from the two nesting periods (Figure S3B–D). Due to some lost samples in the micronutrient analysis, the resulting sample size was small (N = 3 for G1 and N = 3 for G2). No significant differences were found for any of the micronutrients (Table S1).

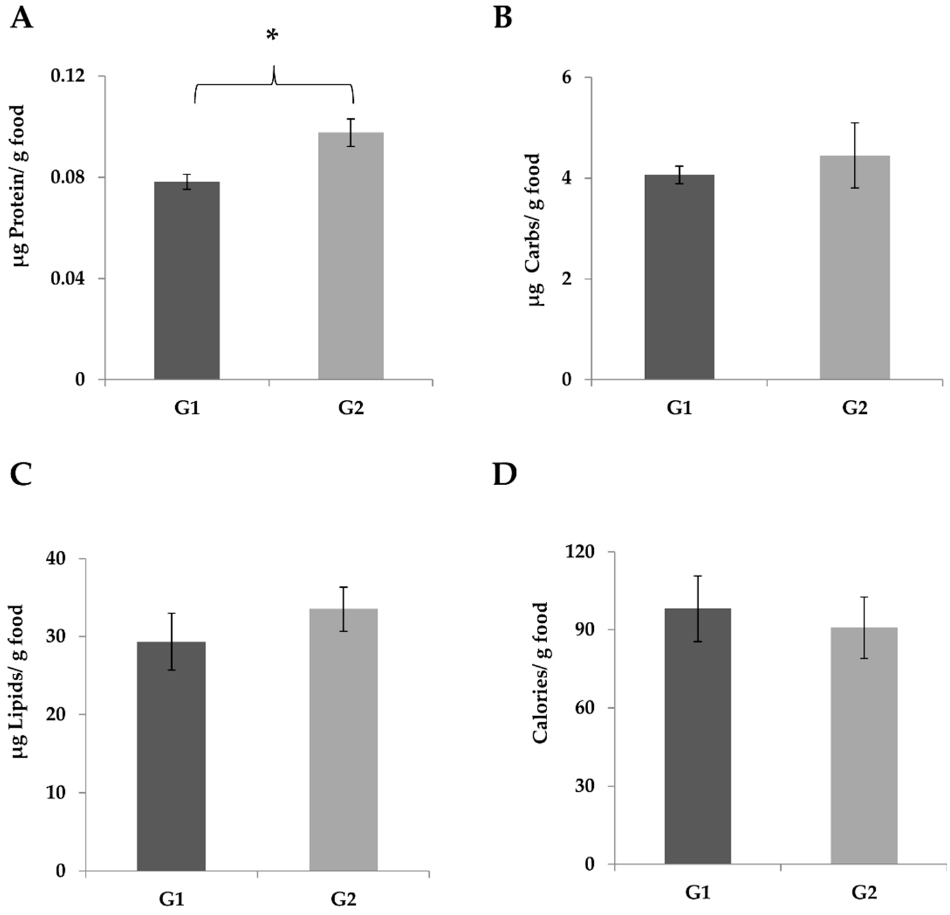

Figure S3. The mean levels of (**A**) protein; (**B**) carbohydrates; (**C**) lipids; and (**D**) calories per unit wet mass in the food taken from individual cells of nests of *Trypoxylon lactitarse* from the G1 and G2 generations. Error bars indicate standard error. For each graph, different letters indicate significant differences between groups (Mann–Whitney U test). Graphs with brackets and an asterisk indicate significant differences.

Table S1. Mean (SD) levels of micronutrients in the food from G1 and G2 generations. (N = 3 for each generation).

Nutrient	G1 µg/g Food	G2 µg/g Food
Ca	1.33 (0.16)	0.96 (0.31)
Cu	0.12 (0.016)	0.095 (0.025)
Fe	0.096 (0.019)	0.22 (0.17)
K	12.16 (2.06)	10.82 (0.93)
Mg	1.40 (0.27)	1.25 (0.36)
Mn	0.053 (0.0085)	0.070 (0.032)
Na	4.52 (0.63)	3.96 (0.0)
Zn	0.18 (0.018)	0.16 (0.018)

Results S2: PCA Results

Table S2. Eigenvalues and percent variance for principle component analysis (PCA) of macronutrient PCN scores.

PC	Eigenvalue	% Variance
1	5.98763	68.693
2	2.19387	25.169
3	0.535058	6.138

Table S3. Loadings for the three principle components of the macronutrient PCN scores.

PCN	PC1	PC2	PC3
Protein	0.012191	0.99565	0.09233
Carbohydrates	0.98667	0.003005	−0.16268
Lipids	0.16225	−0.09308	0.98235

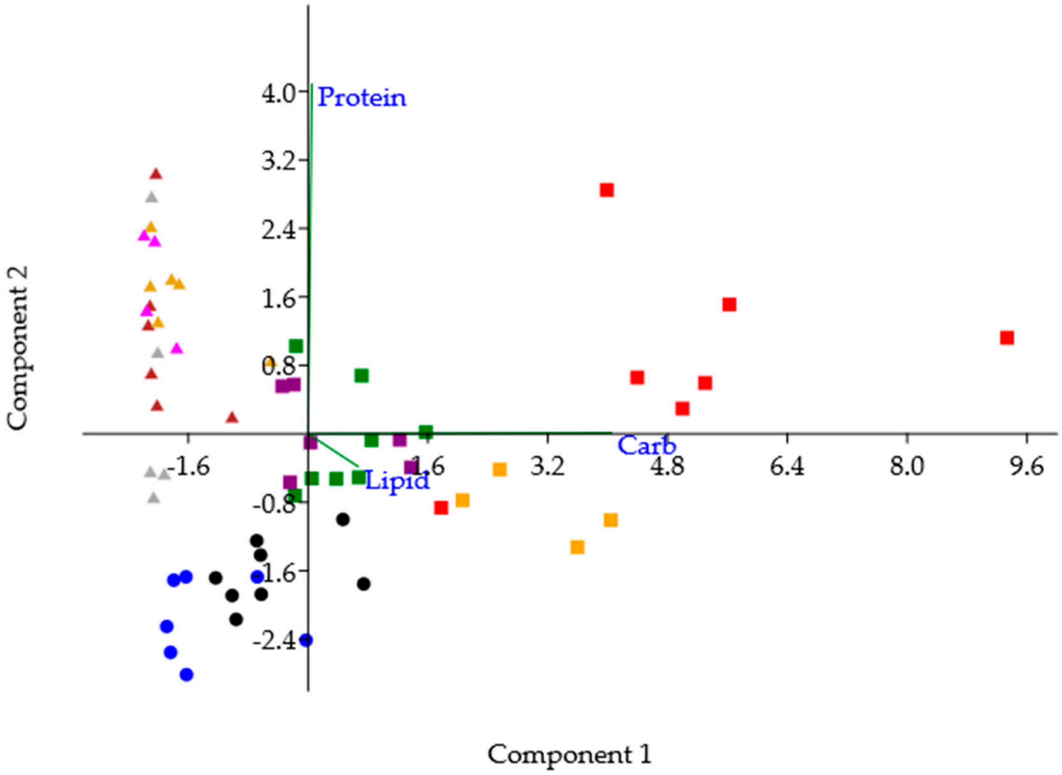

Figure S4. Plots of the first two principle components for percent change in macronutrients (PCN) scores for larvae, pupae, and adults for G1 and G2. The circles indicate larva (black = G1, blue = G2), squares indicate pupae (red = G1 males, green = G1 females, yellow = G2 males, purple = G2 females), and tringles indicate adults (gold = G1 males, brown = G1 females, fuchsia = G2 males, gray = G2 females). Vectors for each nutrient are also shown.

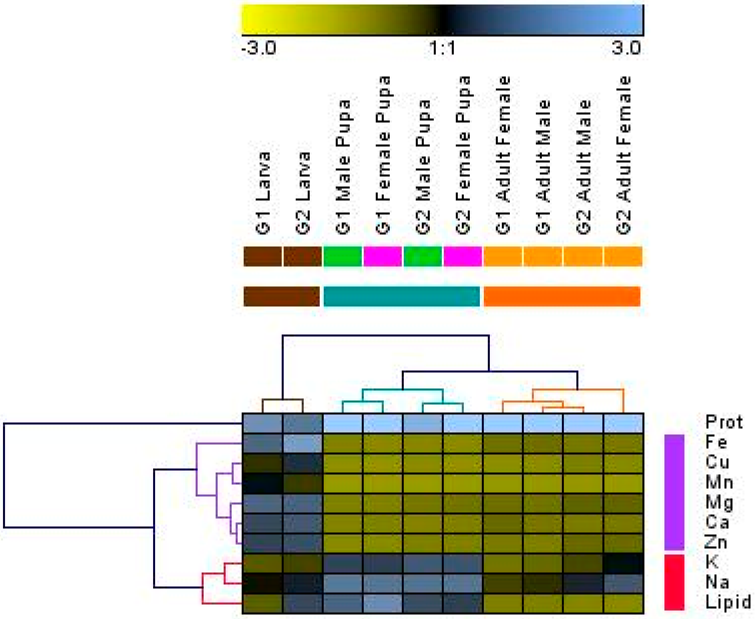

Figure S5. Mean percent change of nutrients relative to initial nutrients (PCN). Increases in PCN are indicated by levels of blue and decreases in PCN are indicated by yellow. Results for the hierarchal analysis are shown for the nutrients and the life stages. Carbohydrates were removed from this analysis to test if male and female pupae would cluster together.

Table S4. Eigenvalues and percent variance for PCA of micronutrient PCN scores.

PC	Eigenvalue	% Variance
1	1.64082	76.142
2	0.313473	14.547
3	0.144585	6.7095
4	0.020251	0.93975
5	0.01406	0.65246
6	0.009699	0.45008
7	0.007566	0.35109
8	0.004489	0.20829

Table S5. Loadings for the first three principle components of the micronutrient PCN scores.

PCN	PC1	PC2	PC3
Ca	0.32289	−0.02884	0.32653
Cu	0.26197	−0.00149	0.3411
Fe	0.71051	0.29149	−0.62464
K	−0.11667	0.5838	0.17846
Mg	0.34973	−0.02117	0.45008
Mn	0.28555	0.058955	0.15168
Na	−0.15978	0.75172	0.1465
Zn	0.2816	−0.06611	0.32858

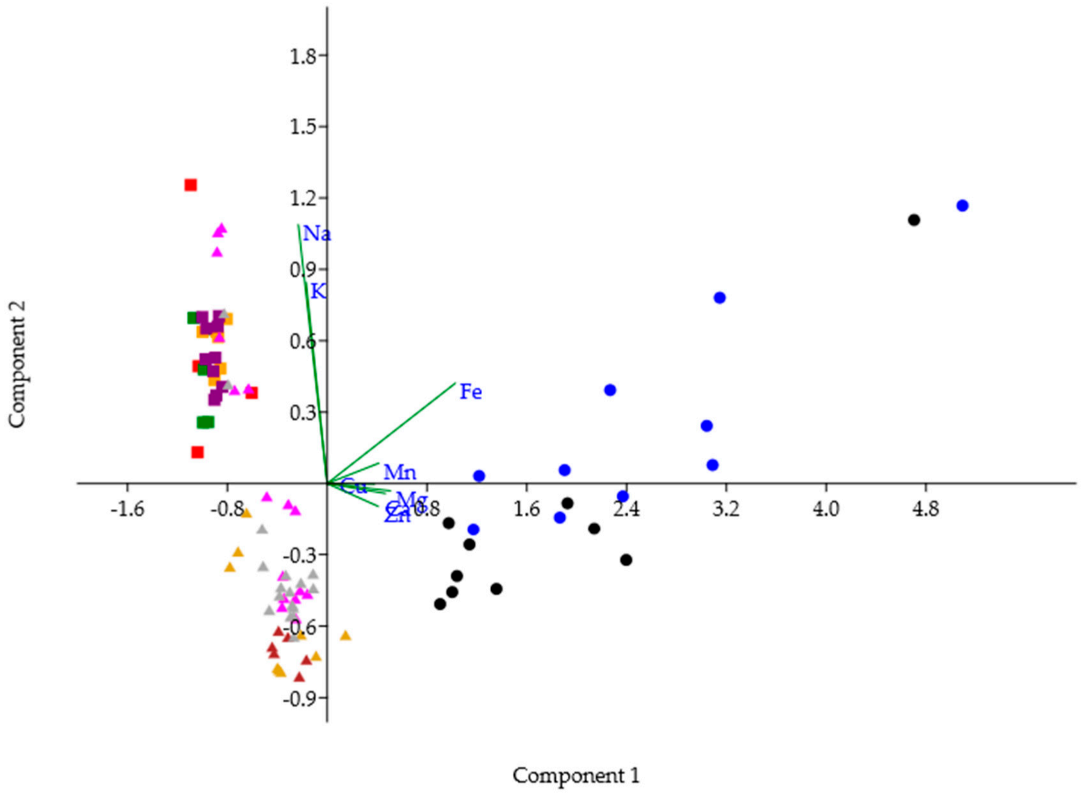

Figure S6. Plot of the first two principle components for percent change in micronutrients (PCN) scores for larvae, pupae, and adults for G1 and G2. The circles indicate larva (black = G1, blue = G2), squares indicate pupae (red = G1 males, green = G1 females, yellow = G2 males, purple = G2 females), and tringles indicate adults (gold = G1 males, brown = G1 females, fuchsia = G2 males, gray = G2 females). Vectors for each nutrient are also shown.

Results S3: CCA Results

Canonical Correlation Analysis for Pupae

Table S6. Eigenvalues and percent variance for PCA of morphology scores (Morph) for female and male analyses.

PC	Females		Males	
	Eigenvalue	% Variance	Eigenvalue	% Variance
Morph1	0.0150	90.30	0.0061	68.48
Morph2	0.0016	9.70	0.0027	31.52

Table S7. Loadings for the three principle components of the morphology scores (Morph) for female and male pupae analyses.

Feature	Females		Males	
	Morph1	Morph2	Morph1	Morph2
Head Width	0.999	0.006	0.995	0.099
Pronotum Width	−0.006	0.999	−0.099	0.995

Table S8. Eigenvalues and percent variance for PCA of macronutrient per unit mass scores (Nutr) for female and male pupae analyses.

PC	Females		Males	
	Eigenvalue	% Variance	Eigenvalue	% Variance
Nutr1	0.0898	57.00	0.1106	70.24
Nutr2	0.0659	41.83	0.0450	28.57
Nutr3	0.0018	1.17	0.0019	0.12

Table S9. Loadings for the three principle components of the macronutrients per unit mass scores (Nutr) for female and male pupae analyses.

Nutrient	Females			Males		
	Nutr1	Nutr2	Nutr3	Nutr1	Nutr2	Nutr3
Protein	−0.069	−0.006	0.998	0.052	0.150	0.987
Carbs	−0.004	0.999	0.006	0.336	0.928	−0.159
Lipids	0.998	0.004	0.069	0.940	−0.340	0.003

Table S10. Results of the canonical correlation analysis for female and male pupae analyses.

	Females			Males		
	Eigenvalue	% Variance	*p*	Eigenvalue	% Variance	*p*
1	33.434	96.88	0.0004	58.254	77.87	0.0003
2	1.077	3.12	0.11	16.551	22.13	0.0032

Table S11. Canonical scores of Nutr and first two with scores for both female and male pupae analyses.

PC	Females		Males	
	With 1	With 2	With 1	With 2
Nutr1	0.913	0.273	0.749	0.663
Nutr2	0.178	0.402	−0.326	0.368
Nutr3	−0.367	0.874	−0.578	0.652

Table S12. Correlations of Morph scores with the first two with scores for both female and male pupae analyses.

PC	Females		Males	
	With 1	With 2	With 1	With 2
Morph1	1.000	−0.001	0.998	−0.058
Morph2	−0.001	−1.000	−0.058	−0.998

5. Canonical Correlation Analysis for Adults

Table S13. Eigenvalues and percent variance for PCA of morphology scores (Morph) for adult female and male analyses.

PC	Females		Males	
	Eigenvalue	% Variance	Eigenvalue	% Variance
Morph1	0.0276	83.22	0.0212	89.87
Morph2	0.0050	14.97	0.0021	8.70
Morph3	0.0006	1.80	0.0003	1.43

Table S14. Loadings for the three principle components of the morphology scores (Morph) for adult female and male analyses.

Feature	Females			Males		
	**Morph1**	**Morph2**	**Morph3**	**Morph1**	**Morph2**	**Morph3**
Head Width	0.989	−0.128	−0.059	0.984	−0.172	0.044
Pronotum Width	0.114	0.483	0.867	0.152	0.688	−0.710
Wing Length	0.082	0.865	−0.493	0.092	0.705	0.703

Table S15. Eigenvalues and percent variance for PCA of macronutrient per unit mass scores (Nutr) for adult female and male analyses.

PC	Females		Males	
	**Eigenvalue**	**% Variance**	**Eigenvalue**	**% Variance**
Nutr1	0.5746	85.06	0.0212	98.05
Nutr2	0.0979	14.99	0.0021	1.86
Nutr3	0.0031	0.45	0.0003	0.09

Table S16. Loadings for the three principle components of the macronutrients per unit mass scores (Nutr) for adult female and male analyses.

Nutrient	Females			Males		
	**Nutr1**	**Nutr2**	**Nutr3**	**Nutr1**	**Nutr2**	**Nutr3**
Protein	−0.048	0.090	0.995	−0.033	−0.020	0.999
Carbs	0.970	−0.236	0.067	0.989	−0.146	0.029
Lipids	0.236	0.968	−0.076	0.145	0.989	0.024

Table S17. Results of the canonical correlation analysis for adult female and male analyses.

	Females			Males		
	**Eigenvalue**	**% Variance**	***p***	**Eigenvalue**	**% Variance**	***p***
1	40.583	94.28	0.0001	19.089	94.89	0.0001
2	2.452	5.70	0.045	0.9937	4.94	0.0004
3	0.009	0.02	0.49	0.0353	0.02	0.70

Table S18. Canonical scores of Nutr and first two with scores for both female and male analyses.

PC	Females		Males	
	**With 1**	**With 2**	**With 1**	**With 2**
Nutr1	0.821	0.103	0.896	0.134
Nutr2	0.414	−0.785	0.366	0.318
Nutr3	−0.393	−0.611	−0.252	0.939

Table S19. Correlations of Morph scores with the first two with scores for both female and male analyses.

PC	Females		Males	
	**With 1**	**With 2**	**With 1**	**With 2**
Morph1	0.988	0.003	0.999	−0.040
Morph2	−0.013	0.739	−0.040	−0.975
Morph3	0.017	0.405	0.005	0.217

© 2017 by the authors; licensee MDPI, Basel, Switzerland. This article is an open access article distributed under the terms and conditions of the Creative Commons by Attribution (CC-BY) license (http://creativecommons.org/licenses/by/4.0/).
